# Identification of the Allosteric Regulatory Site of Insulysin

**DOI:** 10.1371/journal.pone.0020864

**Published:** 2011-06-24

**Authors:** Nicholas Noinaj, Sonia K. Bhasin, Eun Suk Song, Kirsten E. Scoggin, Maria A. Juliano, Luiz Juliano, Louis B. Hersh, David W. Rodgers

**Affiliations:** 1 Department of Molecular and Cellular Biochemistry and Center for Structural Biology, University of Kentucky, Lexington, Kentucky, United States of America; 2 Department of Biophysics, Escola Paulista de Medicina, Universidade Federal de Sao Paulo, Sao Paulo, Brazil; University of Canterbury, New Zealand

## Abstract

**Background:**

Insulin degrading enzyme (IDE) is responsible for the metabolism of insulin and plays a role in clearance of the Aβ peptide associated with Alzheimer's disease. Unlike most proteolytic enzymes, IDE, which consists of four structurally related domains and exists primarily as a dimer, exhibits allosteric kinetics, being activated by both small substrate peptides and polyphosphates such as ATP.

**Principal Findings:**

The crystal structure of a catalytically compromised mutant of IDE has electron density for peptide ligands bound at the active site in domain 1 and a distal site in domain 2. Mutating residues in the distal site eliminates allosteric kinetics and activation by a small peptide, as well as greatly reducing activation by ATP, demonstrating that this site plays a key role in allostery. Comparison of the peptide bound IDE structure (using a low activity E111F IDE mutant) with unliganded wild type IDE shows a change in the interface between two halves of the clamshell-like molecule, which may enhance enzyme activity by altering the equilibrium between closed and open conformations. In addition, changes in the dimer interface suggest a basis for communication between subunits.

**Conclusions/Significance:**

Our findings indicate that a region remote from the active site mediates allosteric activation of insulysin by peptides. Activation may involve a small conformational change that weakens the interface between two halves of the enzyme.

## Introduction

Insulysin (EC 3.4.24.56; also referred to as insulin-degrading enzyme or IDE) is a zinc metallopeptidase in the M16 family that has been extensively studied because of its role in cellular insulin degradation [1] and amyloid beta peptide catabolism [2], [3]. The importance of IDE in regulating insulin metabolism became evident from studies of the GK rat model of type II diabetes mellitus [4] where variants of IDE with reduced catalytic activity were found to be associated with elevated insulin levels. In addition, mice with homozygous deletions of IDE show a marked decrease in insulin degradation [5], while IDE polymorphisms have been associated with type 2 diabetes in humans [6]. Recently, IDE has received considerable attention for its role in the degradation of amyloid beta peptide [5], [7], [8], and a number of studies have provided evidence for a genetic link between IDE and Alzheimer's disease [9], [10], [11], [12]. IDE can degrade many bioactive peptides of varying size in addition to insulin and the amyloid beta peptide, although its role in regulating the levels other peptides *in vivo* has not been established [13], [14].

IDE exists predominantly as a dimer in equilibrium with tetramers and to a lesser extent monomers [15], [16]. It is unique among the enzymes comprising the zinc metallopeptidase M16 family in that it exhibits allosteric kinetic behavior with substrate peptides increasing its activity [16], [17], [18], [19], [20]. In addition, IDE contains a distinct regulatory site first reported by Camberos and coworkers [21] that binds nucleoside triphosphates and alters IDE activity. This site was shown to be distinct from the active site and to primarily interact with the triphosphate moiety [15]. Binding at this site was shown to increase the rate of hydrolysis of some, but not all, IDE substrates [15], [22].

Crystal structures of human IDE (hIDE) complexed with peptides [23], [24], [25], [26] show that the enzyme consists of four structurally related domains that adopt a clamshell-like structure enclosing a large central chamber. Although the four domains have the same overall fold, only the N-terminal domain, domain 1, has an intact active site characteristic of zinc metallopeptidases. Substrate binding requires a conformational change that opens the central chamber, making internal binding sites accessible. Substrates bind at the active site in a manner consistent with other zinc metallopeptidases, but larger peptides can also interact with a distal binding site located in a separate domain of the enzyme. The unliganded structure of hIDE was found to be in the same closed conformation first observed in the peptide complex structures, further indicating that the rate-limiting step for catalysis is the conformational switch from the closed to open state [27].

We report here the crystal structures of rat IDE (rIDE) and a catalytically compromised mutant form, rIDE-E111F. Surprisingly, the structure of the mutant enzyme shows peptide bound at both active and distal sites. Probing the function of the distal site by mutagenesis reveals its key role in the allosteric behavior of IDE. Comparison of the rIDE and rIDE-E111F structures shows small conformational changes upon peptide binding that likely mediate allosteric activation.

## Results

### Overview of the rIDE Structure

The structures of wild type and E111F rIDE are very similar to the published structures of peptide bound and unliganded human IDE (hIDE) [23], [24], [25], [26]. An alignment of wild type rIDE and hIDE from the amyloid beta peptide complex (Protein Data Bank code 2G47) yields an r.m.s.d. of 0.6 Å for 954 Cα atoms. The four structurally similar domains each have an α+β fold and arrange to form the same closed clamshell conformation as hIDE, with the N- and C-terminal halves of the clamshell connected by a single polypeptide chain. In the closed conformation, the two halves of the clamshell are in contact with one another forming an enclosed cavity, and the active site of the enzyme is accessible from this internal substrate-binding chamber. The two rIDE structures show similar local variations in isotropic atomic displacement factors (B factors), although the values for the peptide bound enzyme are generally higher, particularly for the first two domains (Fig. S1).

### Location and Identification of Ligands Bound to rIDE-E111F

**Figure 1:**
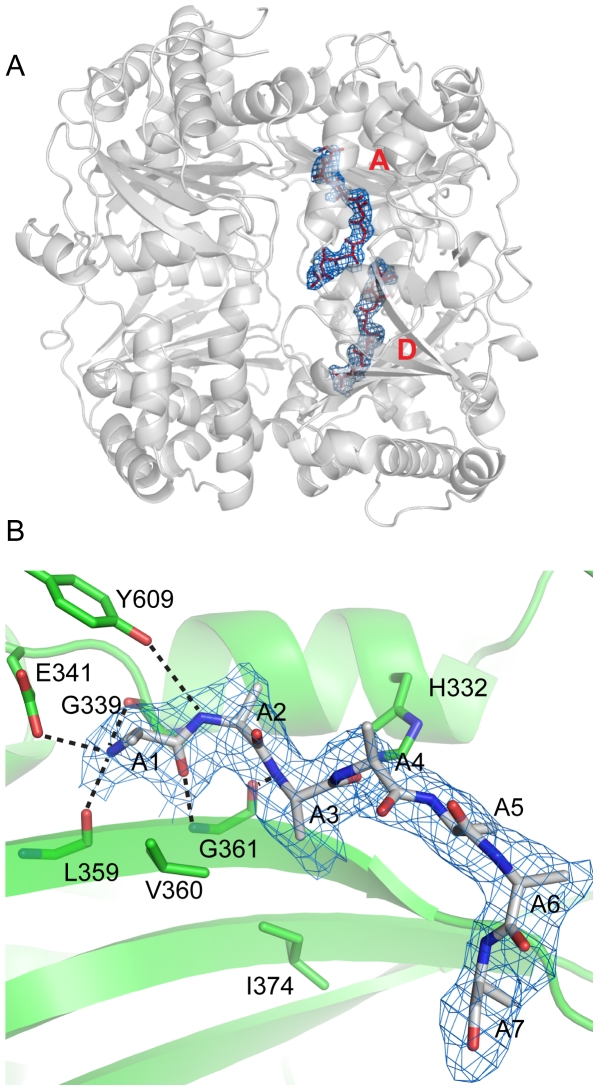
The rIDE-E111F crystal structure. (A) Overview of the structure. A ribbons representation of the enzyme is shown together with polyalanine ligands (red sticks) built into difference density (Fo-Fc, blue mesh, 2.0 sigma contour) at the active (A) and distal (D) sites. Difference density was produced with phases from a refined model of the enzyme prior to adding peptide models and so is not biased by the peptide models. (B) Interaction of bound peptide with the domain 2 distal site of rIDE-E111F. The polyalanine peptide ligand is shown (residues 1–4 of the seven residues in the model) in a stick representation with difference density calculated as described above (blue mesh, 2.0 sigma contour). Likely hydrogen bonds to the peptide backbone are indicated by dashed lines.

The E111F mutation greatly reduces catalytic activity while maintaining the ability to bind substrates [18]. We began work with this mutant with the intention of using it to obtain structural information on a variety of complexes between the enzyme and substrate peptides. Interestingly, though, a difference (Fo-Fc) map made with the rIDE-E111F data in the absence of added substrate peptides revealed two regions of positive electron density located within the central cavity of the enzyme (Fig. 1a) consistent with bound peptide ligand. One segment of difference density was found adjacent to the active site of the enzyme in domain 1, and the second segment was found in the structurally equivalent region of domain 2.

Two separate polypeptide chains were modeled into the ligand density. The ligands were built as polyalanine, since density for the peptide side chains was weak and fragmented. The first ligand was modeled as an eight-residue peptide bound at the active site, where it is positioned to interact with the enzyme through a series of hydrogen bonds with the peptide backbone (Fig. S2). The second ligand, located at the vestigial active site present in domain 2, the distal site, was modeled as a seven-residue peptide that is also positioned to make a number of hydrogen bond interactions with the enzyme (Fig. 1B). Both peptides adopt an extended conformation, aligning along the last strand of the central sheet of their respective interacting domains consistent with the usual location of substrate peptides in zinc metallopeptidases [28]. For the peptide in the distal site, the main chain carbonyl groups of Gly339 and Leu359 as well as the side chain of Glu341 are in position to interact with the N-terminal amino group. Additionally, the side chains of Tyr609 and H332 and the main chain carbonyl of Gly361 may make hydrogen bonds with the peptide backbone.

**Figure 2:**
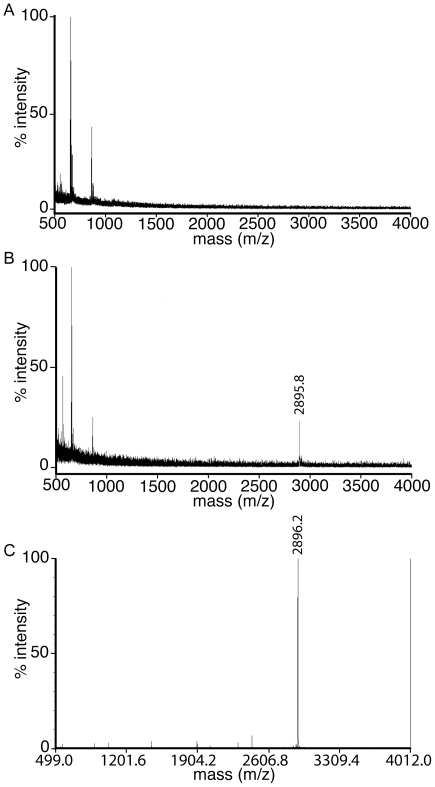
Mass spectrometry identification of ligand bound to rIDE-E111F. (A) MALDI-TOF analysis of purified rIDE-E111F before removal of the polyhistidine fusion sequence. Only matrix peaks are observed. (B) MALDI-TOF analysis of rIDE-E111F after cleavage of the polyhistidine fusion sequence. A peak representing bound ligand is evident. (C) MALDI-TOF analysis of the polyhistidine fusion tag eluted from Ni-NTA agarose resin after normal rIDE-E111F purification. This peak matches the peak representing ligand bound to purified rIDE-E111F in panel B and is consistent with the expected mass of the fusion sequence.

To identify the bound ligand, samples were analyzed by mass spectrometry at various steps during the purification of rIDE-E111F: (1) the enzyme before proteolytic removal of the polyhistidine fusion sequence used in purification, (2) purified enzyme after elution from the Ni-NTA resin by proteolytic removal of the polyhistidine sequence, and (3) imidazole eluate from the metal affinity resin containing any polyhistidine fragment remaining bound after IDE elution. No significant mass peaks consistent with the observed ligands were observed before the cleavage of the polyhistidine fusion sequence (Fig. 2A), suggesting that the unknown ligand was not being co-purified from cell lysates. However, a mass peak at 2895.8 Da was seen in the sample after cleaving the fusion sequence with TEV protease to elute the enzyme from the Ni-NTA resin (Fig. 2B). No such peak was observed for the wild type rIDE purified in the same manner or in a control containing only the TEV protease. A sample containing N-terminal polyhistidine fusion sequence eluted from the Ni-NTA agarose gave a peak at 2896.2 Da (Fig. 2c), closely matching the peak in the enzyme sample. MS/MS analysis of the 2896.2 Da peak gave 18 of 20 expected b-ions (Fig. S3a), 12 of 21 expected y-ions, and at least 3 of 22 expected y*-ions (Fig. S3b), allowing unambiguous identification of the ligand sequence (Fig. S3c). This result is consistent with the bound ligand being the polyhistidine fusion sequence after removal of its N-terminal methionine residue and acetylation of the new N-terminal serine (N-Ac-SYYHHHHHHDYDIPTTENLYFQ, expected mass of 2895.9 Da). Posttranslational modification of this type for polyhistidine fusion sequences of proteins expressed in insect cells has been noted previously [29]. These data indicate that the ligands observed in the crystal structure are the cleaved polyhistidine fusion sequence bound to rIDE-E111F. Given the positions of the two modeled peptides, it is possible that they represent two portions of a single polyhistidine fusion sequence with the joining residues disordered and therefore not visible in the crystal structure. This type of binding at the active and distal sites of IDE has been seen with other peptides [26]. It is also possible, however, that different peptides are bound at the active and distal sites, with only eight of 22 residues ordered in the active site peptide and seven of 22 residues ordered in the distal site peptide. Bound peptides are presumably not seen associated with wild type rIDE because the polyhistidine fusion sequences are degraded by the active enzyme.

### Mutations in the Distal Site Eliminate Allosterism

Our previous work showed that IDE displays allosteric kinetics and that small peptides can act as activators of the enzyme [16], [17], [18], [19], [20]. The finding in this study of peptide ligand bound to the distal site, as well as similar findings by others [24], [26], suggested that this site may be involved in mediating the allosteric regulation of rIDE. To test this hypothesis, we mutated several distal site residues in close proximity to the bound peptide. Three mutant versions of rIDE, Y609F, V360S, and I374S (see Fig. 1) were prepared and the mutant constructs analyzed for allosteric kinetics and activation by a small peptide. The side chain of Y609 is positioned to hydrogen bond to the backbone of bound peptide as noted previously, and V360 and I374 are positioned to interact with side chains of a peptide bound at the distal site.

**Figure 3:**
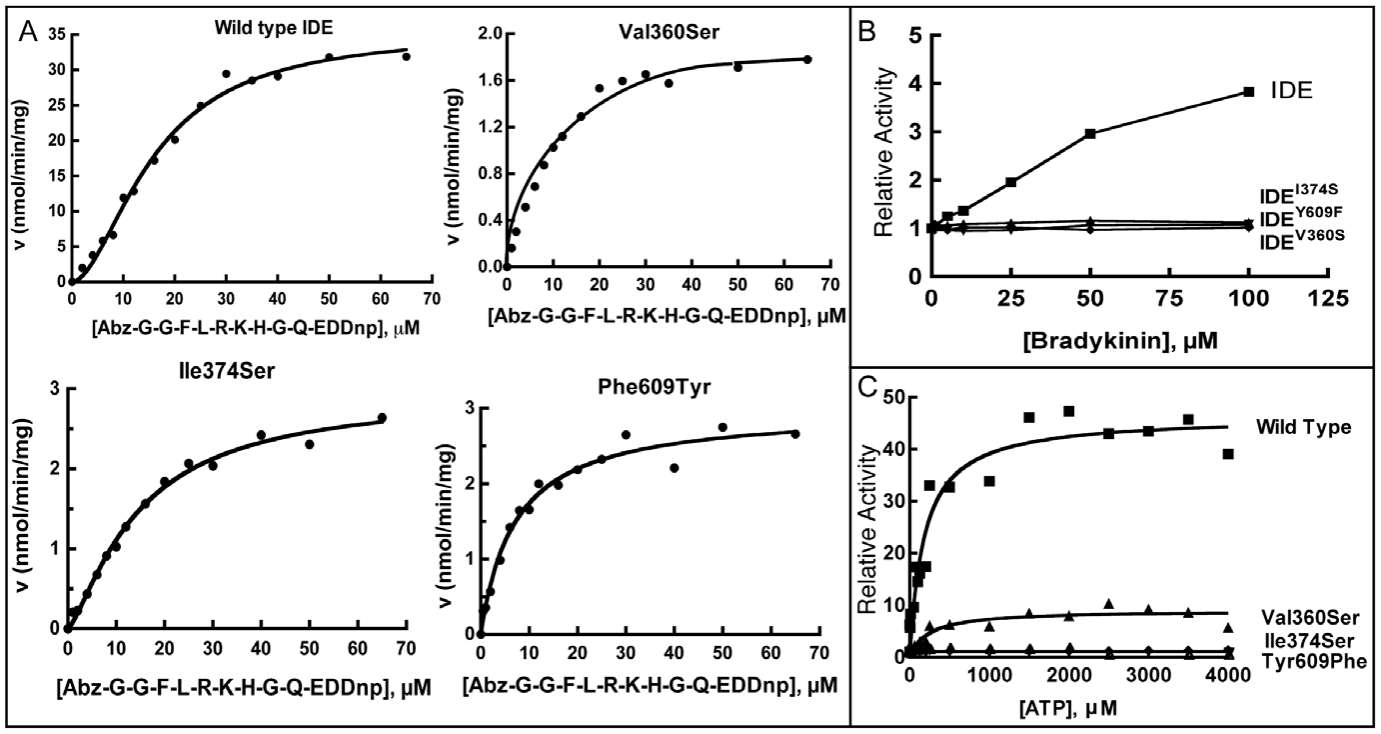
Effects of distal site mutations. (A) Comparison of the kinetics of wild type IDE with the distal site mutants. Activity was measured with varying amounts of Abz-GGFLRKHGQ-EDDnp substrate as indicated. Either hyperbolic (Michaelis-Menten equation) or sigmoidal curves (Hill equation) were fit to the data. (B) Effect of bradykinin on the activity of wild type IDE and the distal site mutants. (C) Activation of wild type IDE and distal site mutants by ATP. Relative activity is the fold activation above the level of the wild type enzyme alone.

Initial velocity versus substrate concentration plots (Fig. 3A) for activity on the fluorogenic peptide substrate Abz-GGFLRKHGQ-EDDnp show that the allostery present in the wild type enzyme is lost in each of the three distal site mutants. Fitting the data with a sigmoidal function gives Hill coefficients (Table 1) of 1.80 for the wild type enzyme and 1.04, 1.07, and 1.12 for the Y609F, V360S, and I374S mutants. V_max_ values for the mutants were all decreased approximately 10 fold relative to wild type, consistent with the loss of activation, while apparent K_m_ values remain roughly the same. Heterotropic activation with the small peptide bradykinin (approximately 4-fold for the wild type enzyme) is also lost in all three mutants (Fig. 3B), showing directly the absence of allosteric activation.

**Table 1:** Kinetics of IDE and distal site mutants.

	Wild Type	Tyr609Phe	Val360Ser	Ile374Ser
Vmax (nmol/min/mg)	35.5±1.6	2.6±0.56	2.01±0.08	3.45±0.22
Hill coefficient	1.8±0.16	1.04±0.14	1.07±0.30	1.12±0.2
Km (µM)	15.87±1.19	6.86±1.18	8.61±0.81	8.5+1.74
K_D_^TNP-ATP^ (µM)	1.0±0.1	2.0±0.3	3.7±0.8	2.1±0.5

**Figure 4:**
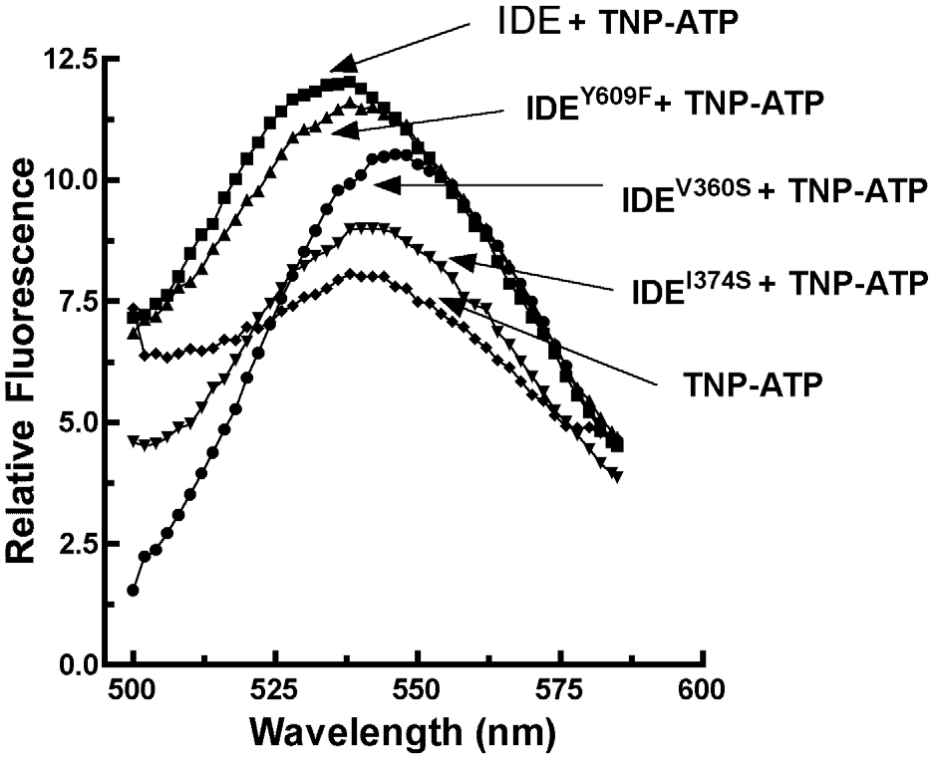
Fluorescence spectra of TNP-ATP bound to wild type IDE and the distal mutants. Fluorescence emission spectra of 10 µM TNP-ATP in 50 µM Tris-HCl, pH 7.4, was measured in the presence of 1.5 µM of each wild type IDE and the indicated IDE distal mutants. Fluorescence spectra were recorded with a λ_exc_ = 403 nm on a Perkin-Elmer LS55 Luminescence Spectrometer.

ATP also acts as a heterotropic activator of IDE, increasing the cleavage rate of the fluorogenic peptide substrate Abz-GGFLRKHGQ-EDDnp more than 40 fold [15]. However, ATP activation of the V360S mutant was reduced to 8 fold, and activation by ATP was not significant for the I374S and Y609F mutants (Fig. 3C). In all three mutants, enhancement of TNP-ATP fluorescence emission indicates that they still bind the ATP analog, although with lower affinity than the wild type enzyme (Table 1, Fig. S4). However, the degree of TNP-ATP fluorescence enhancement is diminished in the mutants, as is the blue shift in the emission peak observed upon binding to the wild type enzyme (Figs. 4). These differences in the emission spectrum of bound TNP-ATP indicate a change in the local environment in the mutants relative to the wild type enzyme, which may result from the loss or alteration of a conformational change associated with allosteric activation.

### Structural Basis for Allosterism

**Figure 5:**
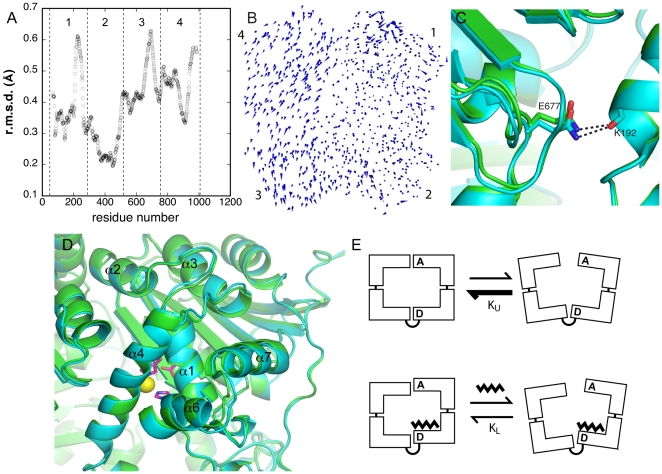
Basis for allosteric activation. (A) Plot of r.m.s. main chain atom positional differences between unliganded IDE and rIDE-E111F with bound peptide ligands at the active and distal sites. Boundaries for domains 1–4 are indicated by the vertical dashed lines. The two structures were superimposed on Cα positions in domains 1 and 2. (B) A porcupine type diagram illustrating the conformational change between unliganded IDE and rIDE-E111F superimposed on domains 1 and 2. Cones indicate the direction and magnitude of differences between Cα positions. Domains are labeled. The size of the cones has been scaled by a factor of 3 relative to the dimensions of IDE to make the conformational change visible in a small diagram. (C) Example of a change in an interface contact between two halves of IDE comparing aligned unliganded IDE (green) and rIDE-E111F (cyan) structures. (D) Conformational changes in the active site domain (domain 1). Superimposed structures of unliganded IDE (green) and rIDE-E111F (cyan) are shown. Residues at the active site are shown in a stick representation, and the zinc ion cofactor is drawn as a yellow sphere. (E) Model of IDE allosteric activation. In the absence of bound peptide at either the active or distal sites, the equilibrium between the closed and open forms of IDE (K_U_) is shifted toward the closed form, which prevents substrate binding and product release. With peptide bound at the distal site (and possibly the active site), the equilibrium (K_L_) shifts to increase the population in the open form, enhancing peptide binding and product release.

Comparison of the ligand free rIDE structure and the rIDE-E111F structure with ligand bound at the distal and active sites suggests a likely structural basis for allosterism. Alignment of the two enzyme structures on Cα positions in the N-terminal halves (domains 1 and 2) of the molecules reveals a difference in the relative positions of the C-terminal portions of the molecule (Fig. 5A, B; SI Movie S1). Binding ligand appears to induce a small shift of domains 3 and 4 relative to domains 1 and 2. While the observed structural rearrangement is small, with a maximum amplitude of 1.5 Å (r.m.s.d. of 0.58 Å for domain 3 and 4 main chain atoms), it is largely concerted, with nearly all Cα positions moving in the same direction (Fig. 5B). Interactions across the interface between the N- and C-terminal halves of the molecule are affected (Table S1, Fig. 5C). The number of residues involved in the interface increases from 133 in the wild type enzyme to 135 in the mutant, although the total solvent accessible surface area buried decreases slightly from 2402 Å^2^ for the wild type to 2324 Å^2^ for the mutant. Overall, both the total number of hydrogen bonds and salt bridges across the interfaces decreases in the peptide bound mutant (wild type: 29 H-bonds, 13 salt bridges; IDE-E111F: 28 H-bonds, 11 salt bridges). It is important to note that while the total number of residues and polar contacts change modestly on peptide binding, there are even more extensive differences in particular residues involved in the interface and those making polar contacts, as is evident on examination of Table S1.

Given the number of changes in the interface between the two halves of the molecule, its stability is also likely altered. Destabilizing the interface would increase partitioning of the enzyme into an open conformation (with the two halves hinging about the chain linking domains 2 and 3) necessary for substrate binding and product release. Since it is believed that the rate of adopting the open conformation limits IDE activity [26], weakening the interface by peptide binding in the distal site (and possibly the active site) would be expected to activate the enzyme. The relative shift of the two halves of the enzyme is also is evident when comparing the liganded and unliganded hIDE structures determined by Tang and coworkers [24], [26], [27], providing additional evidence for the significance of the observation in this study. As already noted, the crystallographic thermal factors for the first two domains of the ligand bound mutant are consistently higher than those for the unliganded enzyme (Fig. S1). This difference may reflect a higher relative mobility of the two halves of the peptide bound enzyme, which would be consistent with a weakening of the interface.

In addition to the largely translational shift between the two halves of the enzyme, other changes in conformation between the unliganded enzyme and rIDE-EIIIF are evident. Elements within the active site domain (domain 1) rotate with respect to the remainder of the domain as well as domains 2–4 (Fig. 5D). The rotation, which occurs roughly about an axis through the domain 1-domain 2 interface, involves helical elements on one side of the central sheet of the domain. In particular, helices 1 (containing the catalytic and two zinc ion binding residues) and 2 (residues 105–135) as well as helices 3–7 (residues 157–247) rotate largely as a rigid body (∼5°). Helices 3–7 are positioned over the active site shielding it from solvent. Since the helical elements that rotate border on the substrate-binding site, it is possible that the change in orientation accompanies peptide binding at the active site in rIDE-E111F. Interestingly, helices 2 and 4 form part of the interface between the two halves of the molecule, largely interacting with elements in domain 4. Thus the conformational change in domain 1 may play a role in the shift in the two halves between the unliganded and ligand-bound IDE structures. Alternatively, the change in the interface might drive the conformational change in the active site domain.

**Figure 6:**
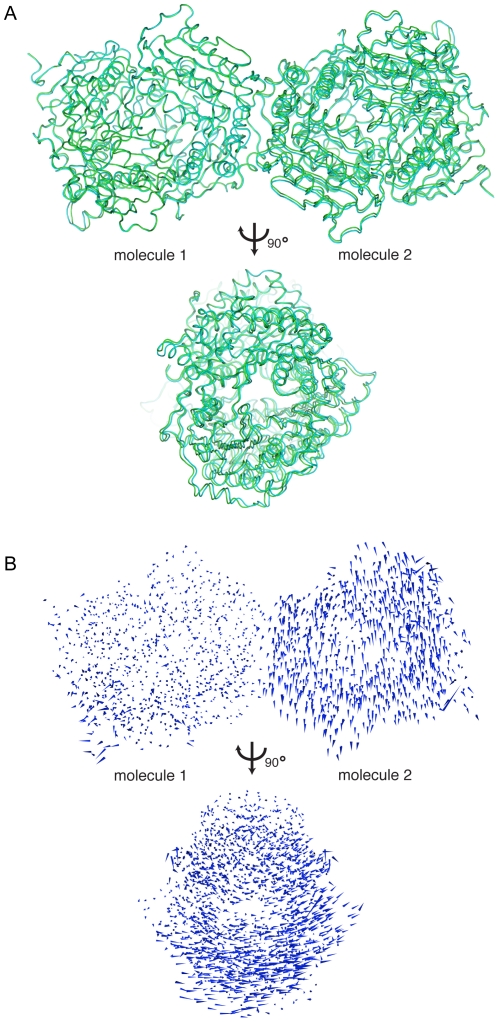
Changes at the dimer interface. (A) Superimposed dimer structures of unliganded IDE (green) and rIDE-E111F with peptide ligand bound at the active and distal sites (cyan). Dimers are aligned based on Cα positions of one monomer (molecule 1). (B) Porcupine type representation of the change in Cα positions between the two structures aligned on molecule 1. Cones represent the magnitude and direction of Cα positional changes. The size of the cones has been scaled by a factor of 2.5 relative to the dimensions of IDE to emphasize the conformational shifts. The orientations of the molecules are the same as those in panel A.

One other conformational change occurs between the unliganded wild type enzyme and rIDE-E111F. IDE functions as a dimer [15], [16], [26], [30], and one of the contacts in the human IDE crystals has been identified as the dimer interface [20], [26]. This interface has also been confirmed as the dimer interface with the rat enzyme [20]. In rIDE-E111F, the subunits undergo a relative rotation compared to the unliganded enzyme (Fig. 6; SI Movie S2). This rotation occurs about an axis roughly orthogonal to the dimer axis, giving maximum shifts of about 2.5 Å when the structures are aligned on one monomer. The changes reduce the number of residues involved in the interface (per monomer) from 45 in the wild type enzyme to 42 in the mutant (residues Pro760, Tyr766, and Phe1005 no longer participating), although the total surface area actually increases slightly from 1392 Å^2^ in the wild type to 1432 Å^2^ in the mutant (Table S2). The pattern of polar contacts is also altered somewhat, with differences at positions 586, 706, 756, 914, 1001, and 1009. In total, the number of interfacial hydrogen bonds changes from 14 in the wild type to 12 in the mutant, and the number of salt bridges remains at four, although different residues are involved. Overall, the conformational changes between the wild type and peptide-bound mutant IDE do not greatly disturb the packing in the crystal (Fig. S5, Table S3).

## Discussion

The results of the present study illuminate the mechanism of allosteric regulation in IDE. The crystal structure of rIDE-E111F shows peptide binding contacts at two distinct locations, the active site in domain 1 and a distal site located in domain 2. Changing the distal site through mutagenesis reduces the activity of the enzyme and converts its kinetics from allosteric to classical Michaelis-Menten. Mutations at the distal site also eliminate the previously observed heterotropic activation produced by small peptide substrates [16] and reduce or eliminate activation by ATP [15].

In earlier work, we noted that the substrate bradykinin acts as an activator of the reaction of IDE with Abz- GGFLRKHGQ-EDDnp, while other peptide substrates, such as β-endorphin and dynorphin A(1–17), show activation at low concentrations and inhibition at higher concentrations [16]. This can be explained by their relative affinity for the distal site versus the active site. The higher the affinity for the distal site relative to the active site the more the peptide acts as a pure allosteric activator, while binding at the active site produces competitive inhibition. It is interesting to note that the crystal structure of IDE in the presence of bradykinin [24] shows peptide bound only at the distal site. This observation is consistent with bradykinin having a higher affinity for the distal site than the active site and therefore acting as a pure activator.

IDE crystallizes in a closed conformation in which the active site and substrate binding surfaces are inaccessible, and it seems likely that it is present primarily in the closed form in solution. The enzyme therefore must undergo a hinge-like motion to an open conformation in order to bind substrate and release products [26], [27]. Tang and coworkers [26], [27] have shown that destabilizing the interface between the N- and C-terminal halves of IDE by mutagenesis increases its activity, indicating that adopting an open conformation is rate limiting. The conformational change induced by peptide binding seen in this study suggests that allosteric activation occurs by destabilizing the N- and C-terminal domain interface. For small substrates, peptide binding at the distal site increases the population in the open conformation, enhancing both substrate binding and product release. Larger substrates can bind at both the active and distal sites simultaneously, and the enzyme does not therefore exhibit allosteric kinetics or heterotropic activation with these molecules [16].

Since IDE functions as a dimer [16], [26], [30], a relevant question is whether activation occurs within or between subunits. Free monomeric IDE does not show allosteric kinetics and is not activated by added small peptides, indicating that the dimer is required for allostery [20]. At first glance, these results would seem to suggest that activation occurs across the dimer interface. However, work with mixed dimers carrying combinations of mutations at the active or distal sites in one or both subunits demonstrates that the primary allosteric effect occurs within each subunit of the dimer [18], [31]. We therefore favor a model in which binding of peptide to the distal site induces a conformational change in the same monomer that shifts the equilibrium toward the open form (Fig. 5E). The conformational change is, however, dependent on the monomer participating in dimer contacts with its partner subunit. Without this interaction, peptide binding at the distal site does not greatly alter the equilibrium between open and closed forms. A full scheme diagramming this model for IDE activation is given in Fig. S6.

How does peptide binding drive alteration of the interface between monomer halves? At the distal site, bound peptide lies close to the hinge region connecting the two halves of the molecule. It also interacts with Tyr609 from domain 3, which is located on the opposite side of the hinge. Binding of peptide in this region might influence the conformation of the hinge region and surrounding interface surfaces by direct interactions with residues on either side of the hinge. It is also possible that more general electrostatic effects, for example peptide binding changing the charge-charge interactions between the halves of the molecule, play a role in altering the interface.

Although activation within a monomer likely accounts for IDE allostery, previous data with mixed dimers indicate that some communication between the subunits occurs [16]. In particular, mutations at the active site of one monomer appear to affect the activity of the other monomer, and it is possible that this occurs in the E111F mutant. The observed conformational shift at the dimer interface between ligand free wild type enzyme and ligand bound rIDE-E111F reported here suggests a possible structural basis for this communication. The conformational change in the active site domain (domain 1) of rIDE-E111F observed in this study may induce changes in the dimer interface, which in turn cause corresponding changes in the active site of the other subunit. Since the active site domains of the two subunits do not participate in the dimer contacts, conformational changes may be transmitted through domain 4, which is in close contact with the active site domain and forms a large part of the dimer interface. If so, the changes in that domain are too subtle to be discerned by comparing the two structures described in this report. It should be noted that there is some evidence that bound ligands affect the oligomerization state of IDE [15], [16], [18]. The possibility remains open, therefore, that the conformational shifts observed in this study changes the affinity of the dimer interface and affect activity by altering oligomerization. We also note that the absence of the metal ion in the peptide bound mutant enzyme could potentially play a role in altering conformation. However, loss of the metal ion in zinc metallopeptidases has been found to not alter structure [32], and it seems unlikely that it is influencing the observed differences in conformation.

It has previously been established that polyanions, including ATP, strongly activate IDE toward hydrolysis of small peptides by binding to a site distinct from the active site [15]. The finding that mutations in the distal site greatly reduce or eliminate activation by ATP implies a mechanistic linkage between the two forms of activation. A difference in the bound TNP-ATP emission spectrum caused by mutations in the distal site indicates that changes at that site affect the environment of the ATP binding site. The distal site does not have a concentration of basic residues and therefore seems an unlikely location for ATP binding, suggesting that the effect of mutations there may propagate to another site on the enzyme. One possibility is that the interface between the N- and C-terminal halves of IDE is affected by ATP binding and that mutations at the distal site affect the ability of bound ATP to induce these changes. Further structural studies will be needed to explore this interesting link between the two activating sites.

Identification of the allosteric site of IDE and possible mechanisms of activation lays the groundwork for further investigations into the activity of this unusual and important enzyme. In addition, this work provides a framework for attempts to develop selective activators of IDE or modified versions of the enzyme that may be used as therapeutics for the treatment of Alzheimer's disease and other disorders.

## Materials and Methods

### IDE Expression and Purification

Native rat IDE (rIDE, residues 42–1016, 95% identical and 98% similar to the human ortholog), E111F-rIDE, and IDE distal site mutants were expressed as hexahistidine fusion proteins in SF9 insect cells [18], [33]. All enzymes produced begin at methionine 42, which is believed to be the *in vivo* start site [34], and extend through the native C-terminal residue leucine 1019. Selenomethionine incorporation [35] was achieved by pre-incubating Sf9 insect cells with rIDE-baculovirus for 10 hours in methionine-depleted SF-900 II SFM media (Invitrogen). Selenomethionine was then added to the media and the cells were allowed to incubate another 72 hours before harvesting.

IDE was purified (Fig. S7) using HIS-select Ni-NTA agarose (Sigma) utilizing the N-terminal hexahistidine affinity tag (hexahistidine sequence and a linker containing a TEV protease cleavage site), eluting by proteolytic removal of the hexahistidine sequence with TEV protease [18], [33]. Protein was estimated using Coomasssie Blue Reagent (BioRad) with BSA as a standard.

### Preparation of IDE Distal Site Mutants

IDE distal site mutants were generated by site-directed mutagenesis using the QuikChange kit (Stratagene) with the wild type rIDE cDNA in pFastBac HTb as a template. Oligonucleotides used for mutagenesis with the base changes in bold and underlined were:

Y609F:

Forward 5^′ -^ CTCAACGACTATGCA**TTT**GCAGCAGAGCTAGCA-3^′^

Reverse 5^′^ - TGCTAGCTGTGCTGC**AAA**TGCATACTCGTTGAG – 3′

V360S:

Forward 5^′ -^ TGGGTAAACACCCTG**TCT**GGGGGACAGAAGGAA- 3^′^

Reverse 5^′^ – TTCCTTCTGTCCCCC**AGA**CAGGGTGTTTACCCA - 3^′^

I374S:

Forward 5^′ –^ GGTTTTATGTTTTTT**TCC**ATTAATGTGGACTTA- 3^′^

Reverse 5^′^ – TAAGTCCACATTAA**TGG**AAAAAAACATAAAACC- 3^′^

### Crystallization and Structure Determination

Enzyme at 8 mg/ml in 50 mM Tris 7.4, 1 mM DTT, and 100 mM NaCl was crystallized by sitting drop vapor diffusion. Protein was mixed 1∶1 with well solution containing 100 mM sodium citrate pH 6.5, 100 mM ammonium acetate, and 20% PEG 4000, and crystals grown at 20 or 22°C. Full-sized (0.2 mm longest dimension) crystals grew reproducibly within two weeks. Crystals produced in this manner diffracted only to low resolution, but dehydration [36] by brief (1–5 second) transfer to 50% PEG 4000 prior to flash cooling in liquid nitrogen gave high resolution diffraction.

Data were collected at the Southeast Regional Collaborative Access Team (SER-CAT) 22-ID and 22-BM beamlines at the Advanced Photon Source (APS), Argonne National Laboratory. Se-MAD data sets were collected at both SERCAT 22-ID at APS and X25A at the National Synchrotron Light Source (NSLS) through each of their mail-in crystallography programs. X-ray data were processed using HKL2000 [37] and the space group of the crystals found to be C2 with one molecule per asymmetric unit and cell parameters of a = 115.8 Å, b = 71.2 Å, c = 114.6 Å, α = 90.00, β = 92.46, and γ = 90.00 for the wild type enzyme and a = 115.5 Å, b = 71.0 Å, c = 114.4 Å, α = 90.00, β = 92.97, and γ = 90.00 for the E111F mutant. The data sets were checked for possible pseudomerohedral twinning, but no significant twinning was detected using PHENIX XTRIAGE [38].

Molecular replacement was performed using Phaser [39] and CCP4 [40] using the deposited hIDE structure in complex with the amyloid beta peptide (Protein Data Bank code 2G47) as a search model. The Rfree value of the model after molecular replacement was 0.42. Anomalous density maps calculated using phases from selenomethionine data showed peaks consistent with methionine residue positions in the model, confirming the molecular replacement solution. Model building and refinement using the molecular replacement solution was done using COOT [41] and REFMAC5 [42]. Water picking was done using PHENIX [38], [43], [44] with manual editing of all additions in COOT. Six TLS groups, determined using the TLS Motion Server [45], were used in atomic displacement refinement for each structure. Final refinement was performed using CNS [46] and PHENIX. Molecular structure figures were made using PyMOL (http://www.pymol.org). The final wild type model contains residues 42–963 and residues 980–1011 with 232 ordered solvent molecules and one zinc ion. The rIDE-E111F model contains residues 42–965 and 978–1011 with 185 ordered solvent molecules and two peptides, one comprised of seven alanine residues and the other of eight alanine residues. Data and refinement statistics are listed in Table 2 and Ramachandran plots, made with PROCHECK [47], are provided in Fig. S8. Protein-protein interfaces were analyzed using the program PISA [48].

**Table 2:** Summary of crystallographic data and model refinement.

	unliganded IDE	rIDE-E111F
Crystallographic data		
Wavelength (Å)	0.9718	1.0000
Resolution (Å)	50-2.08	50-2.14
Last shell (Å)	2.16-2.08	2.24-2.14
Average redundancy (last shell) (%)	4.6 (2.9)	2.8 (2.0)
*R*_merge_ (last shell) (%)	0.10 (0.47)	0.088 (0.41)
*I/σI* (last shell) (%)	11.9 (2.1)	14.96 (3.98)
Completeness (last shell) (%)	97.5 (87.9)	98.7 (99.6)
Refinement		
Resolution (Å)	50-2.08	50-2.14
Number of reflections included in refinement	52364	47228
*R*_work_/*R*_free_	0.19/0.26	0.19/0.26
estimated coordinate error (Å)^a^	0.31	0.33
r.m.s.d. bond lengths (Å)	0.008	0.009
r.m.s.d. bond angles (°)	1.11	1.16
r.m.s.d. chirality (°)	0.07	0.08
r.m.s.d. planarity (°)	0.005	0.005
r.m.s.d. dihedral angles (°)	14.9	16.4
B^b^ r.m.s.d. bonded atoms	3.8	4.3
Average B for all protein atoms (Å^2^)	39	56
Average B for ordered solvent (Å^2^)	37	48
Number of protein molecules in the asymmetric unit	1	1
Number of protein residues in the asymmetric unit	954	958
Number of peptide residues in the asymmetric unit	0	15
Number of protein and peptide atoms in the asymmetric unit	7802	7909
Solvent content (%) (Matthews coefficient)	41.8 (2.1)	41.7 (2.1)
Number of solvent molecules	232	185
Number of metal ions	1	0

a Maximum likelihood method in Phenix [43], [44].

b Isotropic thermal factor.

### Identification of Bound Ligand

The identity of the unknown ligand observed in the rIDE-E111F structure was determined by MALDI-TOF analysis using a crystallization drop from which crystals were harvested. In addition, a fresh sample of rIDE-E111F was analyzed at specific steps during purification. To verify that the unknown ligand was not introduced with the TEV protease added during purification, a TEV sample was also analyzed as a control. MS/MS analysis was performed using the precursor peak at 2896 from the HIS-tag alone sample in order to determine sequence information. MALDI-TOF and MS/MS experiments (scan range 500–4000) were performed at The Scripps Center for Mass Spectrometry and at The University of Kentucky Proteomics Core Facility. Data analysis was performed using MS-Product [49] and Data Explorer Software (Applied Biosystems).

### Enzyme Activity Assay

The activity of IDE was measured by following the increase in fluorescence upon hydrolysis at the L-R bond of the fluorogenic peptide Abz-GGFLRKHGQ-EDDnp on a SpectraMax Gemini XS fluorescence plate reader using an excitation wavelength of 318 nm and emission wavelength of 419 nm. Reactions were carried out in 200 µl volumes containing 50 mM Tris-HCl, pH 7.4, 1.92 µg of wild type rIDE, 4.6 µg of rIDE-V360S, 4.3 µg rIDE-I374S, or 1.63 µg of rIDE-Y609F. For measurements of activation by bradykinin, IDE activity was determined with 10 µM Abz-GGFLRKHGQ-EDDnp as substrate in 50 mM Tris-HCl buffer, pH 7.4. The amount of protein was 0.5 µg for wild type rIDE and 5 µg for each of the IDE distal-site mutants. Data were analyzed using Softmax 4.0 (Molecular Devices) and kinetic parameters calculated with Prism (Graphpad Software) [50], [51]. Specific activity with the Abz-GGFLRKHGQ-EDDnp substrate is 8,131 nmol/min/mg for the wild type enzyme and 5.2 nmol/min/mg for the E111F mutant [18]. Under these assay conditions, both wild type and mutant rIDE are almost completely dimeric [31].

### Fluorescence Measurements

Fluorescence measurements of TNP-ATP binding were performed on a Perkin-Elmer LS55 Luminescence Spectrometer. Titrations were monitored at an excitation wavelength of 403 nm and emission wavelength of 547 nm. The temperature of the sample was maintained at 20±0.1°C by circulating thermostatically controlled water through the cuvette holder. Assays were conducted in 200 µL reaction mixtures containing 50 mM Tris-HCl, pH, 7.4, 10 µM Abz- GGFLRKHGQ-EDDnp as substrate, and 0.5 µg wild type rIDE, 2.3 µg rIDE-V360S, 2.0 µg rIDE-I374S or 1.62 µg rIDE-Y609F and the indicated concentration of ATP. The fluorescence of TNP-ATP was subtracted from the total fluorescence of enzyme+TNP-ATP to yield specific fluorescence enhancement (ΔF).

## Supporting Information

Figure S1 Isotropic thermal factors from rIDE crystal structures. The isotropic thermal factors for each residue (average of main chain atoms) are plotted versus residue number. Values for the wild type unliganded IDE model are in filled circles, and values for the E111F IDE mutant with bound peptides are in open triangles.
(TIF)

Figure S2 Interaction of bound peptide with the active site (domain 1). The polyalanine peptide is shown as a stick representation in difference density (blue mesh, 2.0 sigma contour). Hydrogen bonds to backbone groups in residues 1, 5, 6,and 7 are indicated by dashed lines.
(TIF)

Figure S3 Identity of ligand bound to rIDE-E111F. (A) MS/MS analysis of the precursor peak 2896.2017 (*) with observed b-ion peaks labeled. (B) MS/MS analysis of the precursor peak 2896.2017 (*) with observed y-ion and y*-ion peaks labeled. (C) Schematic of the expected b- and y-ions which may be observed upon fragmentation of the HIS-tag with MS/MS analysis (top). Summary of the results from the MS/MS analysis of the 2896.2017 precursor peak (bottom; np indicated no peaks were observed).
(TIF)

Figure S4 ATP analog binding to wild type and distal site mutant IDE. The fluorescence increase on binding of (2,4,6-trinitrophenyl)ATP (TNP-ATP) is plotted for the indicated IDE constructs as a function of ligand concentration. Data were fit to a hyperbolic one site binding mechanism.
(TIF)

Figure S5 Packing in the rIDE crystals. Molecules in a unit cell (black outline) are shown as C trace worms in two orthogonal views for the wild type unliganded and E111F mutant-peptide complex crystals. Molecules used to generate symmetry mates are shown in green and cyan for the wild type unliganded IDE and E111F mutant-peptide complex, respectively. Symmetry related molecules are shown in blue, wild type, and orange, liganded mutant.
(TIF)

Figure S6 Allosteric mechanism of IDE. A mechanistic model based on functional and structural observations is given. The two halves (domains 1 and 2, and domains 3 and 4) of an IDE monomer are shown schematically in closed and open forms with schematic representations of bound substrates and ligands. The active (A) and distal (D) sites are labeled. Substrate peptide is shown as a narrow zigzag line, and allosteric peptide as a heavy zigzag line. In the absence of bound ligand, the closed form of the molecule predominates (equilibrium K_1_). Binding substrate shifts the population distribution even more toward the closed form (K_3_) because the peptide interacts with both halves of the enzyme. The subsequent cleavage step is fast, but product release is likely rate limiting, since the closed form is still strongly favored (K_4_). Binding of peptide at the distal site alters the interface to increase the proportion of enzyme in the open conformation (K_6_). Substrate binding (K_7_) is therefore likely enhanced. Importantly, product release is also enhanced by the shift in distribution toward the open conformation (K_9_), effectively activating the enzyme at the substrate concentrations used for assays.
(TIF)

Figure S7 Enzyme purification. A Coomassie-stained SDS polyacrylamide gel is shown with wild type or mutant enzyme at different stages of purification: lanes 1 and 5, crude lysate; lanes 2 and 6, flow through from nickel affinity column; lanes 3 and 7, 20 mM imidazole elution; lanes 4 and 8, 200 mM imidazole elution (final purity).
(TIF)

Figure S8 Crystal structure main chain torsion angle plots. Ramachandran plots of main chain phi and psi angles are shown for wild type unliganded (A) and E111F mutant-peptide complex (B). Summary statistics are shown beside each plot.
(TIF)

Table S1 Interface between the N- and C- terminal halves of wild type and peptide bound E111F mutant IDE.
(PDF)

Table S2 Dimer interface of wild type and peptide bound E111F mutant IDE.
(PDF)

Table S3 Crystal contact interfaces of wild type and peptide bound E111F mutant IDE.
(PDF)

Movie S1 Monomer conformational change. The enzyme is shown in a backbone worm representation converting from the wild type unliganded form to the rIDE-E111F liganded form. The orientation is the same as that in Fig. 1A.
(MOV)

Movie S2 Dimer conformational change. The enzyme dimer is shown in a backbone representation converting from the wild type unliganded form to the rIDE-E111F liganded form. The orientation is the same as that in the upper image of Fig. S1 panel A.
(MOV)
