# Supplementary figures and images for: Identification of the Allosteric Regulatory Site of Insulysin
Source: PLoS One. 2011 Jun 24;6(6):e20864. doi: 10.1371/journal.pone.0020864 (PMC3123307; doi:10.1371/journal.pone.0020864)

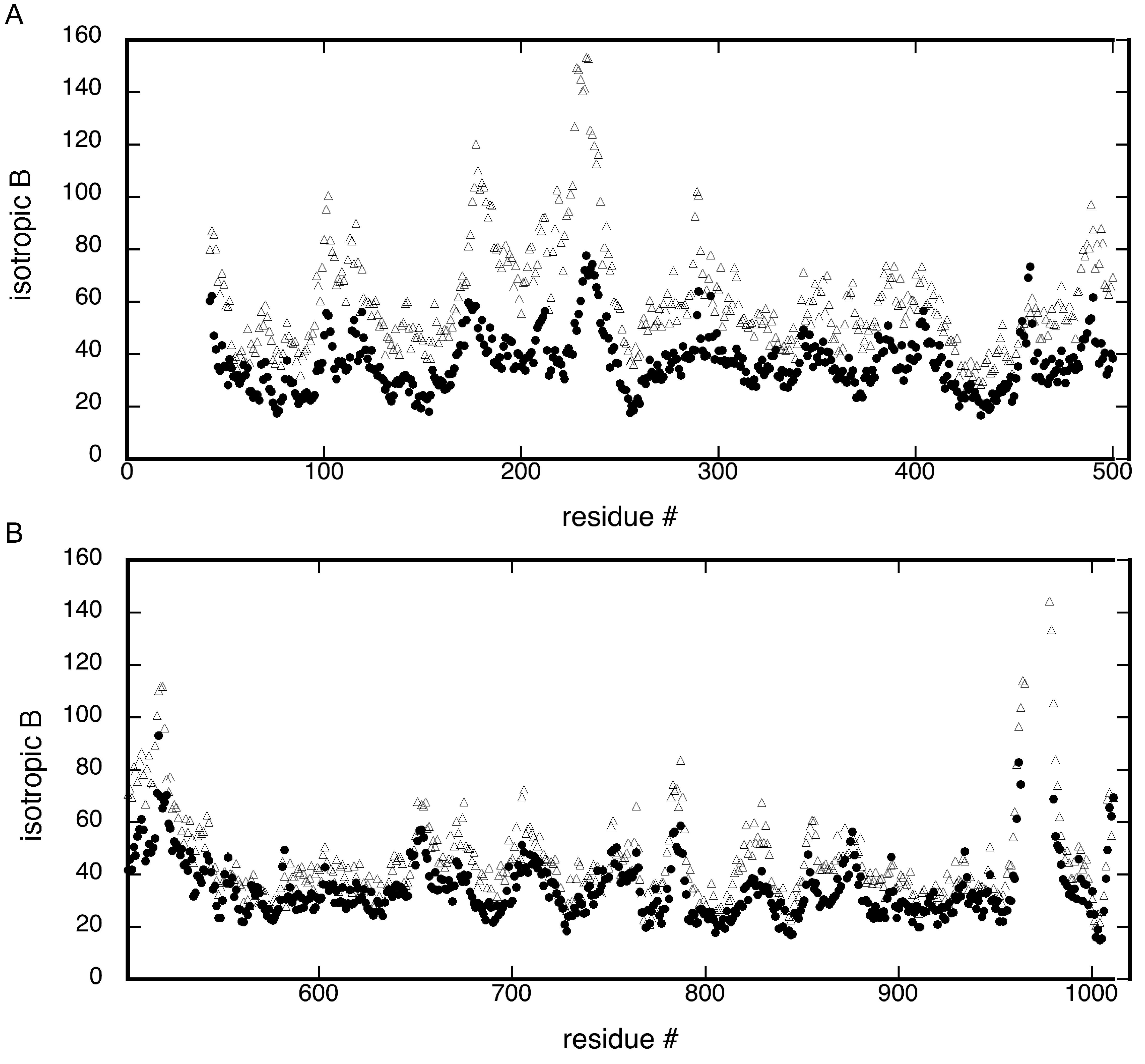

Supplement: Figure S1 — Isotropic thermal factors from rIDE crystal structures. The isotropic thermal factors for each residue (average of main chain atoms) are plotted versus residue number. Values for the wild type unliganded IDE model are in filled circles, and values for the E111F IDE mutant with bound peptides are in open triangles. (TIF) [file pone.0020864.s001.tif]

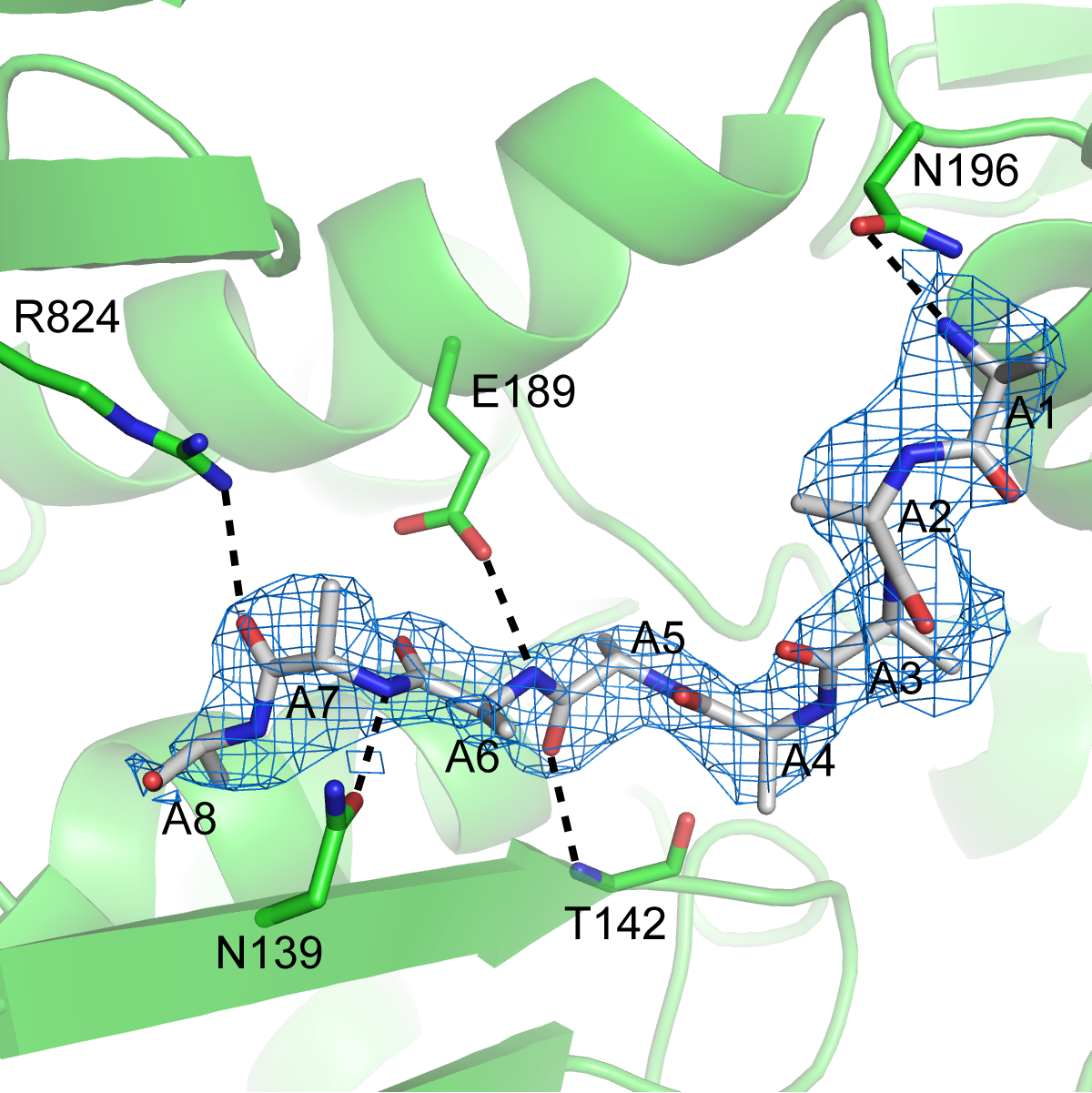

Supplement: Figure S2 — Interaction of bound peptide with the active site (domain 1). The polyalanine peptide is shown as a stick representation in difference density (blue mesh, 2.0 sigma contour). Hydrogen bonds to backbone groups in residues 1, 5, 6,and 7 are indicated by dashed lines. (TIF) [file pone.0020864.s002.tif]

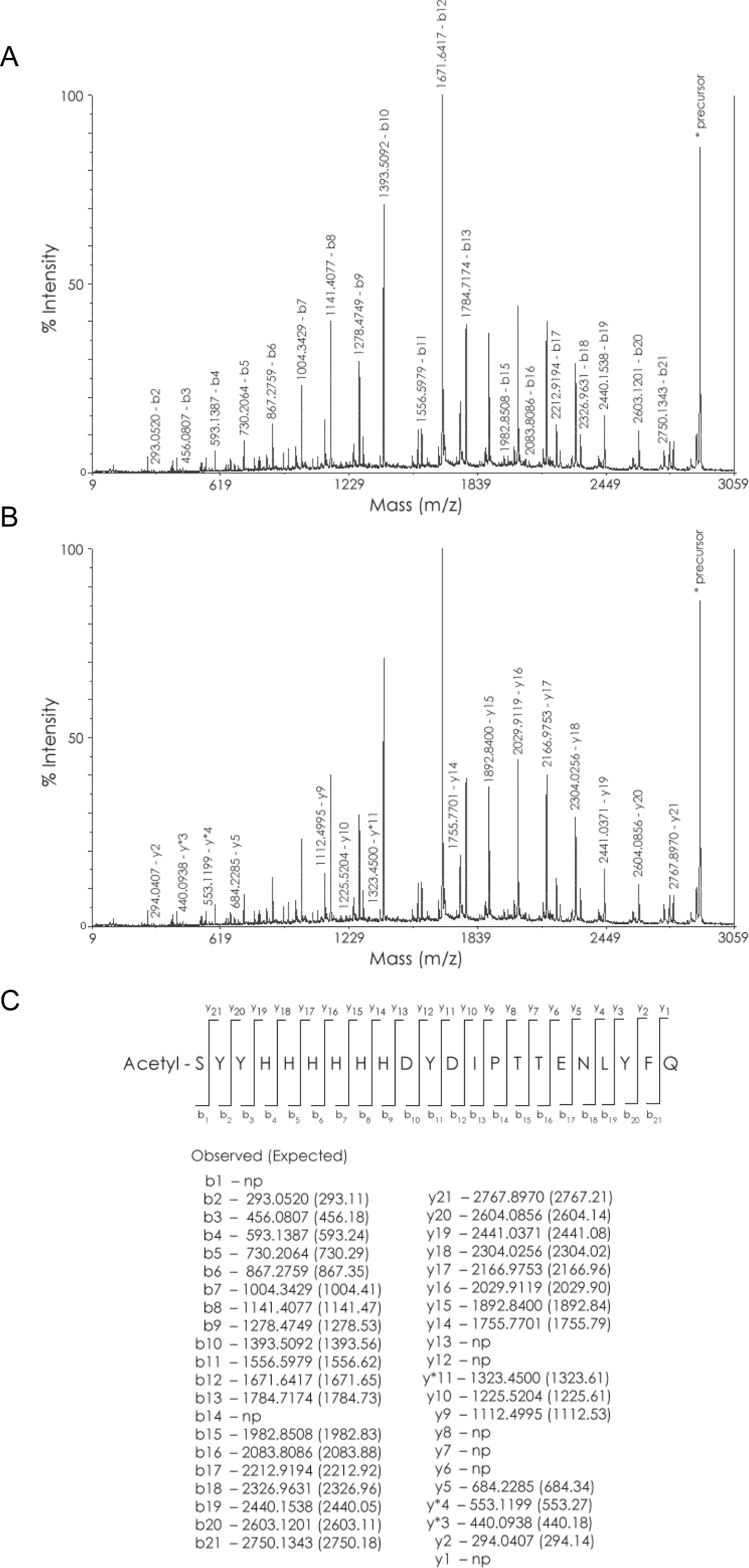

Supplement: Figure S3 — Identity of ligand bound to rIDE-E111F. (A) MS/MS analysis of the precursor peak 2896.2017 (*) with observed b-ion peaks labeled. (B) MS/MS analysis of the precursor peak 2896.2017 (*) with observed y-ion and y*-ion peaks labeled. (C) Schematic of the expected b- and y-ions which may be observed upon fragmentation of the HIS-tag with MS/MS analysis (top). Summary of the results from the MS/MS analysis of the 2896.2017 precursor peak (bottom; np indicated no peaks were observed). (TIF) [file pone.0020864.s003.tif]

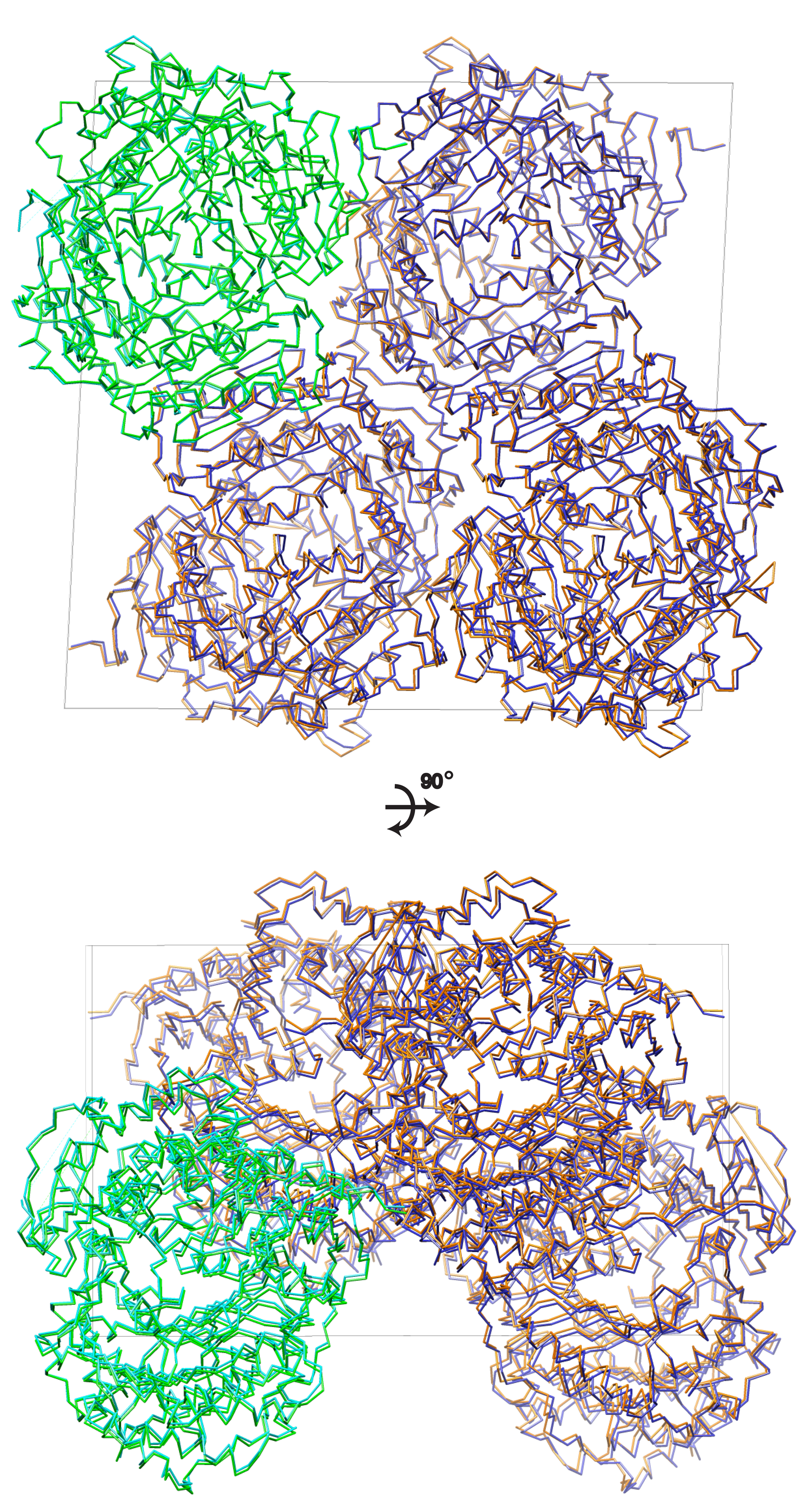

Supplement: Figure S5 — Packing in the rIDE crystals. Molecules in a unit cell (black outline) are shown as C trace worms in two orthogonal views for the wild type unliganded and E111F mutant-peptide complex crystals. Molecules used to generate symmetry mates are shown in green and cyan for the wild type unliganded IDE and E111F mutant-peptide complex, respectively. Symmetry related molecules are shown in blue, wild type, and orange, liganded mutant. (TIF) [file pone.0020864.s005.tif]

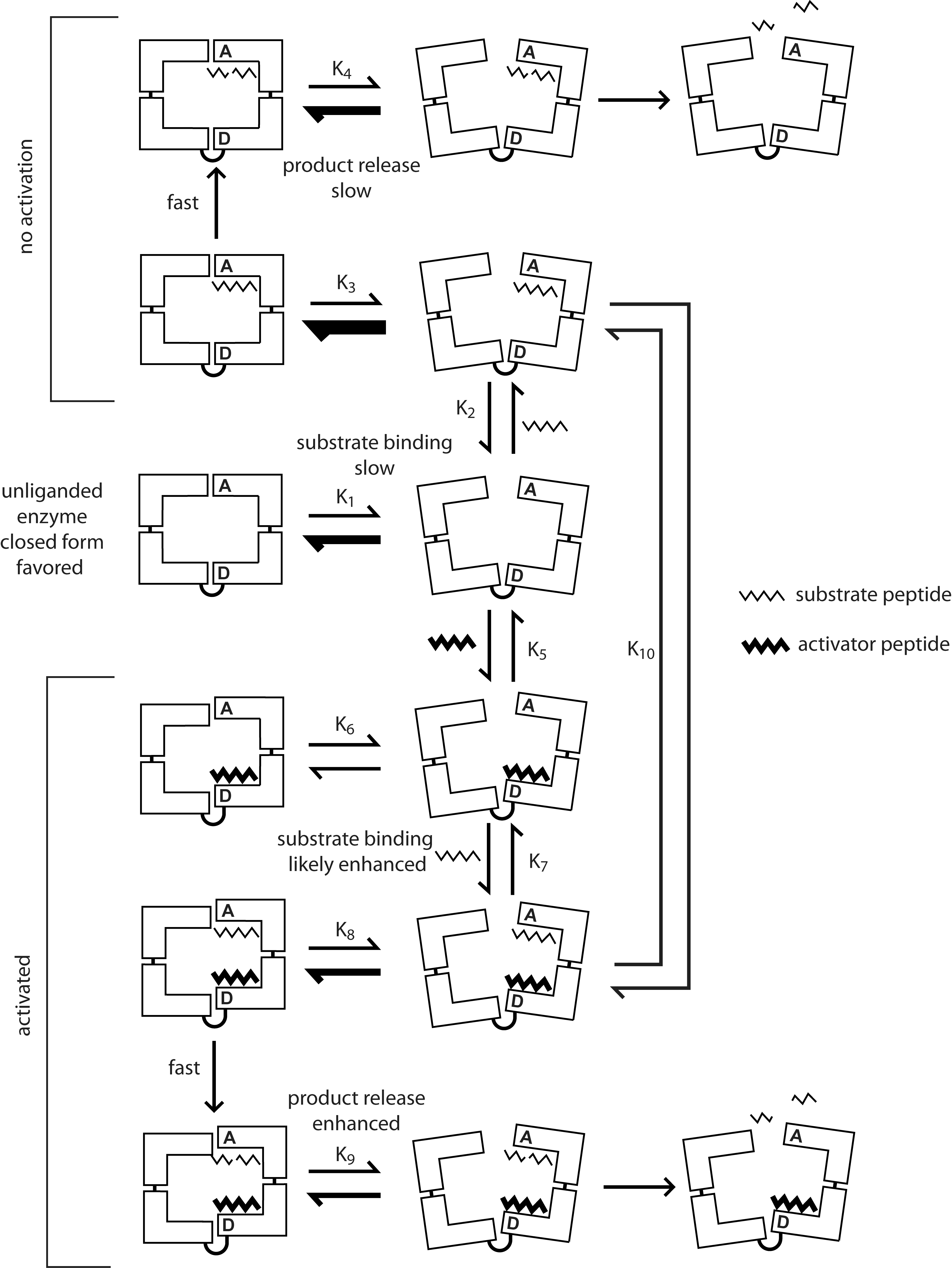

Supplement: Figure S6 — Allosteric mechanism of IDE. A mechanistic model based on functional and structural observations is given. The two halves (domains 1 and 2, and domains 3 and 4) of an IDE monomer are shown schematically in closed and open forms with schematic representations of bound substrates and ligands. The active (A) and distal (D) sites are labeled. Substrate peptide is shown as a narrow zigzag line, and allosteric peptide as a heavy zigzag line. In the absence of bound ligand, the closed form of the molecule predominates (equilibrium K1). Binding substrate shifts the population distribution even more toward the closed form (K3) because the peptide interacts with both halves of the enzyme. The subsequent cleavage step is fast, but product release is likely rate limiting, since the closed form is still strongly favored (K4). Binding of peptide at the distal site alters the interface to increase the proportion of enzyme in the open conformation (K6). Substrate binding (K7) is therefore likely enhanced. Importantly, product release is also enhanced by the shift in distribution toward the open conformation (K9), effectively activating the enzyme at the substrate concentrations used for assays. (TIF) [file pone.0020864.s006.tif]

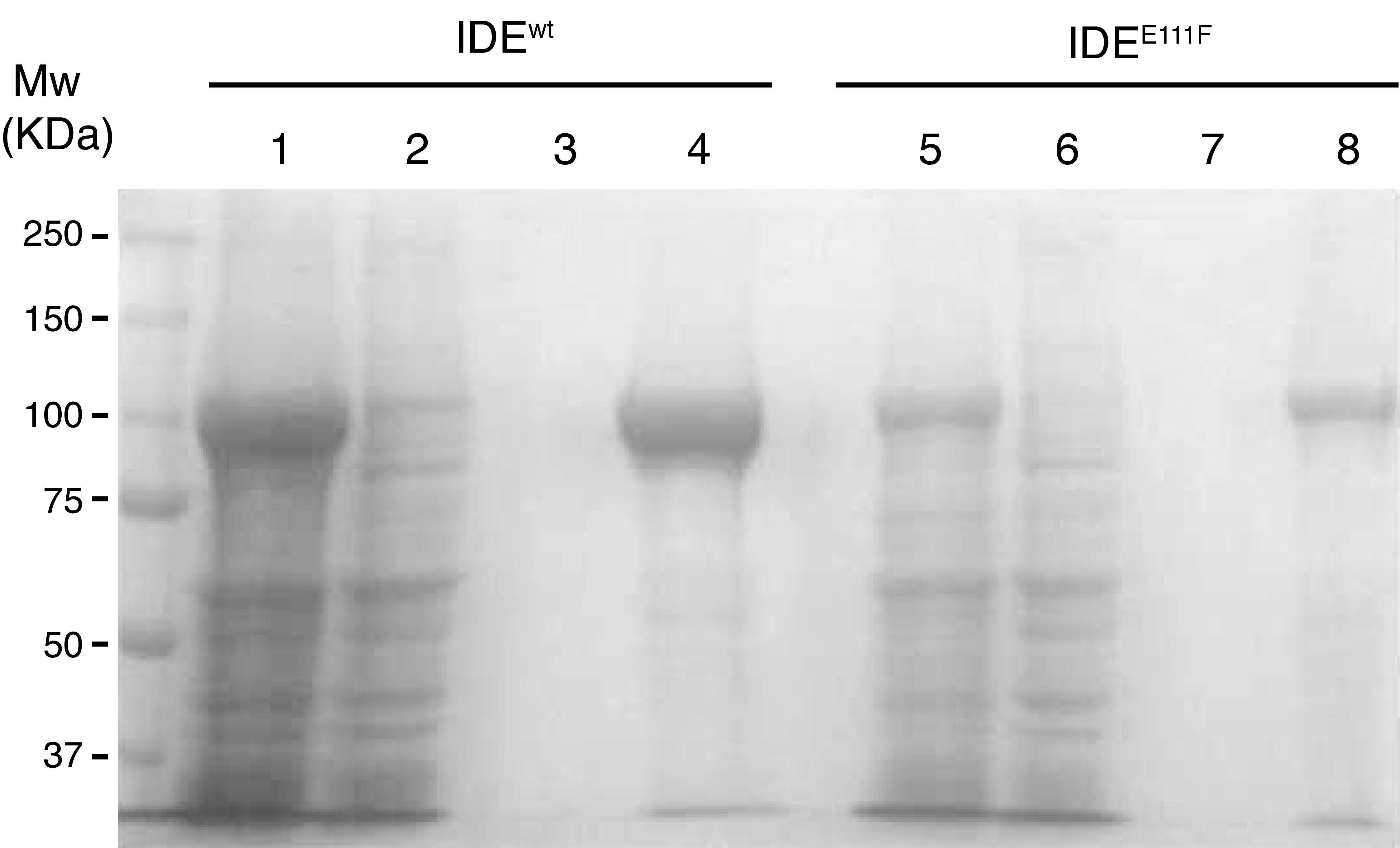

Supplement: Figure S7 — Enzyme purification. A Coomassie-stained SDS polyacrylamide gel is shown with wild type or mutant enzyme at different stages of purification: lanes 1 and 5, crude lysate; lanes 2 and 6, flow through from nickel affinity column; lanes 3 and 7, 20 mM imidazole elution; lanes 4 and 8, 200 mM imidazole elution (final purity). (TIF) [file pone.0020864.s007.tif]

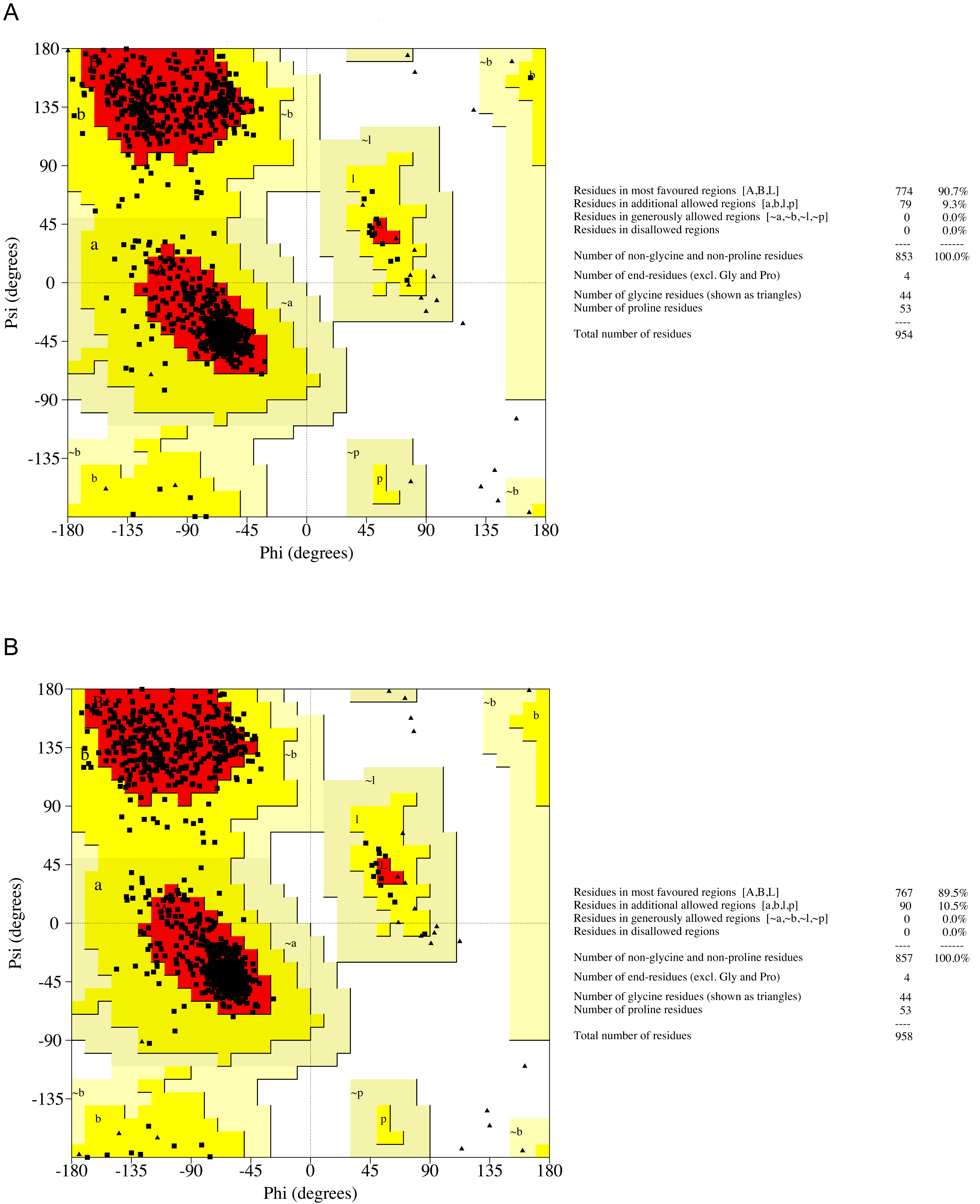

Supplement: Figure S8 — Crystal structure main chain torsion angle plots. Ramachandran plots of main chain phi and psi angles are shown for wild type unliganded (A) and E111F mutant-peptide complex (B). Summary statistics are shown beside each plot. (TIF) [file pone.0020864.s008.tif]
