# Supplementary material for: Identification of the Allosteric Regulatory Site of Insulysin
Source: PLoS One. 2011 Jun 24;6(6):e20864. doi: 10.1371/journal.pone.0020864 (PMC3123307; doi:10.1371/journal.pone.0020864)
Supplement: Table S1 — Interface between the N- and C- terminal halves of wild type and peptide bound E111F mutant IDE. (PDF) [file pone.0020864.s009.pdf]

Table S1. Interface between the N- and C- terminal halves of wild type and peptide bound E111F mutant IDE.

	wild type		E111F mutant	
surface area (Å^2)	2402		2324	
number of interface residues	133		135	
# H bonds	29		28	
# salt bridges	13		11	
residues at the domain interface				
difference wt v. mutant		H bond/salt bridge[a]		H bond/salt bridge[a]
	Glu59		Glu59	
	Thr83		Thr83	
	Asp84	H	Asp84	H
	Lys85	S	Lys85	S
	His112		His112	
	Leu116		Leu116	
Δ	Glu124	HS	Glu124	
Δ	Asn125	H	Asn125	
	Ser128	H	Ser128	H
	Gln129		Gln129	
	Ser132	H	Ser132	H
	Glu133		Glu133	
	His134		His134	
Δ	Als135	H	Als135	
Δ	Gly136		Gly136	H
	Ser137		Ser137	
	Ser154		Ser154	
Δ			Glu156	
	His157		His157	
Δ	Arg181		Arg181	H
Δ	Glu182	H	Glu182	
	Asn184		Asn184	
	Ala185	H	Ala185	H
	Ser188	H	Ser188	H
	Glu189		Glu189	
	Glu191		Glu191	

	Lys192	H	Lys192	H
Δ	Met195		Met195	H
	Asn196		Asn196	
	Ile307		Ile307	
Δ	Lys308	H	Lys308	S
	Asp309	H	Asp309	H
	Ile310		Ile310	
Δ	Arg311	S	Arg311	HS
	His336		His336	
	Gly339	H	Gly339	H
	His340		His340	
Δ	Glu341	H	Glu341	
	Gly342		Gly342	
	Pro343		Pro343	
Δ			Gly344	
	Leu347		Leu347	
	Ser348	H	Ser348	H
Δ	Lys351	H	Lys351	HS
Δ	Ser352		Ser352	H
Δ	Lys353		Lys353	H
	Gly354		Gly354	
	Val356		Val356	
	Asn357	H	Asn357	H
	Asp378		Asp378	
	Leu379		Leu379	
	Thr380		Thr380	
	Glu381		Glu381	
	Glu413	HS	Glu413	HS
	Asp416		Asp416	
	Leu417		Leu417	
	Val420		Val420	
	Als421		Als421	
Δ			Arg423	
	Phe424	H	Phe424	H
	Lys425		Lys425	
	Asp426	HS	Asp426	HS
	Lys427		Lys427	
	Glu428		Glu428	
	Arg429		Arg429	
	Pro430		Pro430	
Δ	Lys483			
	Thr526	H	Thr526	H
	Lys527	HS	Lys527	HS
	Asn528	HS	Asn528	HS
	Glu529	HS	Glu529	HS

	Ile531		Ile531	
	Pro532		Pro532	
	Phe535		Phe535	
	Ile537		Ile537	
	Phe568		Phe568	
	Leu569		Leu569	
	Pro570		Pro570	
	Lys571	HS	Lys571	HS
Δ	Asp602	HS	Asp602	S
Δ	Asn605	H	Asn605	
	Glu606	H	Glu606	H
	Tyr607		Tyr607	
	Tyr609	H	Tyr609	H
	Ala610		Ala610	
	Leu613		Leu613	
	Ala614		Ala614	
	Tyr634		Tyr634	
	Lys657	H	Lys657	H
	Arg658	H	Arg658	H
	Ile661		Ile661	
	Ile662		Ile662	
Δ	Glu664	S	Glu664	HS
	Ala665		Ala665	
	Arg668	H	Arg668	H
	Asn671		Asn671	
Δ	Asn672	H	Asn672	H
Δ	Phe673			
Δ	Arg674			
	Ala675		Ala675	
Δ	Glu676		Glu676	S
	Gln677	H	Gln677	H
	His679		His679	
	Val785		Val785	
	His786		His786	
	Asn787		Asn787	
	Asn788		Asn788	
	Gln813	H	Gln813	H
	Ser816		Ser816	
	Glu817	H	Glu817	H
	Phe820		Phe820	
Δ	Asn821	H	Asn821	
	Arg824		Arg824	
Δ	Thr825		Thr825	H
Δ	Lys826	HS	Lys826	
	Gln828	H	Gln828	H

	Gly830		Gly830	
	Tyr831	H	Tyr831	H
	Ile832		Ile832	
	Gln851		Gln851	
Δ			Ser852	
	Ala881		Ala881	
	Lys884		Lys884	
	His885		His885	
Δ			Gln887	
	Ala888		Ala888	
	Ile891		Ile891	
	Arg892	H	Arg892	H
	Leu894		Leu894	
	Asp895	S	Asp895	S
	Lys896	H	Lys896	H
	Pro897		Pro897	
	Lys898		Lys898	
	Lys899		Lys899	
	Leu900		Leu900	
	Ser901		Ser901	
	Ala902		Ala902	
Δ	Arg961		Arg961	H

[a] Interfaces were characterized with the program PISA [Krissinel E, Henrick K (2007) Inference of macromolecular assemblies from crystalline state. J Mol Biol 372: 774-797].

[b] Only the type, not the number, of interactions is indicated.
