# Supplementary material for: Identification of the Allosteric Regulatory Site of Insulysin
Source: PLoS One. 2011 Jun 24;6(6):e20864. doi: 10.1371/journal.pone.0020864 (PMC3123307; doi:10.1371/journal.pone.0020864)
Supplement: Table S2 — Dimer interface of wild type and peptide bound E111F mutant IDE. (PDF) [file pone.0020864.s010.pdf]

Table S2. Dimer interface of wild type and peptide bound E111F mutant IDE.

	wild type		E111F mutant	
surface area (Å^2)	1392		1432	
number of interface residues	45		42	
# H bonds	14		12	
# salt bridges	4		4	
residues at the dimer interface				
difference wt v. mutant		H bond/salt bridge[b]		H bond/salt bridge[b]
	Pro581		Pro581	
	Phe582		Phe582	
	Val585		Val585	
Δ	Asp586	HS	Asp586	
	Pro587		Pro587	
	Leu588		Leu588	
	His589		His589	
	Glu692		Glu692	
	Val693		Val693	
	Trp695		Trp695	
	Glu699		Glu699	
	Glu702		Glu702	
	Ala703		Ala703	
Δ	Asp706		Asp706	HS
	Arg711		Arg711	
	Ala714		Ala714	
	Phe715		Phe715	
	Gln718		Gln718	
	Arg722	HS	Arg722	HS
Δ	Lys756		Lys756	HS
	Leu758		Leu758	
	Leu759		Leu759	
Δ	Pro760			
	Ser761		Ser761	
	Gln762		Gln762	
	Leu763		Leu763	
	Val764		Val764	

	Arg765		Arg765	
Δ	Tyr766			
	Arg767	H	Arg767	H
	Gln770		Gln770	
Δ	Gln914	H	Gln914	H
	Thr996		Thr996	
	Lys999	H	Lys999	H
	Arg1000	H	Arg1000	H
Δ	Gly1001	H	Gly1001	
	Leu1002	H	Leu1002	H
	Pro1003		Pro1003	
	Leu1004	H	Leu1004	H
Δ	Phe1005			
	Pro1006		Pro1006	
	Leu1007	H	Leu1007	H
	Val1008		Val1008	
Δ	Lys1009	H	Lys1009	
	Pro1010		Pro1010	

[a] Interfaces were characterized with the program PISA [Krissinel E, Henrick K (2007) Inference of macromolecular assemblies from crystalline state. J Mol Biol 372: 774-797].

[b] Only the type, not the number, of interactions is indicated.
