# Supplementary material for: Identification of the Allosteric Regulatory Site of Insulysin
Source: PLoS One. 2011 Jun 24;6(6):e20864. doi: 10.1371/journal.pone.0020864 (PMC3123307; doi:10.1371/journal.pone.0020864)
Supplement: Table S3 — Crystal contact interfaces of wild type and peptide bound E111F mutant IDE. (PDF) [file pone.0020864.s011.pdf]

Table S3. Crystal contact interfaces of wild type and peptide bound E111F mutant IDE.

**interface 2**[a,b]				
	wild type		E111F mutant	
surface area (Å^2)	718		801	
number of interface residues	26		29	
# H bonds	4		6	
# salt bridges	0		2	
residues at interface				
		H bond/salt bridge[b]		H bond/salt bridge[b]
	Asp187			
	Ala207		Ala207	
	Thr208		Thr208	
	Gly209		Gly209	
	Pro211		Pro211	
	Lys223		Lys212	
	Tyr224		Lys223	
	Glu227		Tyr224	H
	Thr228		Thr228	
	Arg229		Arg229	
			Asn231	
	Gln232		Gln232	
			Glu233	H
	Gln294		Gln294	
			Glu295	
	Glu296	H	Glu296	HS
	His297		His297	
	Gln300		Gln300	
	Gly487		Gly487	
	Lys488		Lys488	S
	Thr489		Thr489	
	Asp490	H	Asp490	
	Arg491		Arg491	
	Thr492		Thr492	

			Gln494	
			Gln499	
	Lys501	H	Lys501	H
	Gln502	H	Gln502	H
	Glu503		Glu503	
	Ala504		Ala504	
	Asp508			
		**interface 3**[a,b]		
	wild type		E111F mutant	
surface area (Å^2)	546/565		515/510	
number residues (primary/other molecule)	26/23		22/26	
# H bonds	3		1	
# salt bridges	0		0	
		residues at interface		
		H bond/salt bridge[b]		H bond/salt bridge[b]
	**Lys120**		**Lys120**	
	**Pro122**		**Pro122**	
	**Lys123**		**Lys123**	
	**Glu124**		**Glu124**	
	**Asn125**		**Asn125**	
	**Gln129**		**Gln129**	
	**Asp175**		**Glu133**	
	**Ser177**		**Ser177**	
	**Cys178**			
	**Arg181**		**Arg181**	
	Pro343		Pro343	
	Gly344		Gly344	
	Ser348		Ser348	
	Glu349		Glu349	
	Ser352		Ser352	
	Lys353		Lys353	
	Glu382			
			Asp389	

	Gln399		Gln399	
	Lys400		Lys400	
	Ala403		Als403	
			Lys512	
	Asn515		Asn515	
	Asp517		Asp517	
	Leu518		Leu518	
	Asn519		Asn519	
	Gly520		Gly520	
	Lys521		Lys521	
	Lys523	H	Lys523	
			Leu524	
			Pro525	
	Thr526		Thr526	
	Glu606		Glu606	
			Lys648	
	Glu653			
	Asp655		Asp655	
	Lys656		Lys656	
	Lys657			
			Arg658	
	**Lys826**			
	**Thr869**		**Thr869**	
	**Glu871**			
	**Lys872**	H	**Lys872**	
	**Ala873**		**Ala873**	
	**Glu875**		**Glu875**	
	**Asp876**	H	**Asp876**	H
	**Met877**		**Met877**	
	**Thr878**	H	**Thr878**	
			**Glu879**	
	**Glu880**		**Glu880**	
	**Ala881**		**Ala881**	
	**Asp934**		**Asp934**	
	**Lys938**			
	**Lys941**		**Lys941**	
	**Asp947**		**Asp947**	
			**Asn979**	
	**Glu982**			
**interface 4**[a,b]				
	wild type		E111F mutant	
surface area	559/545		523/540	

(Å^2)				
number residues (primary/other molecule)	22/21		23/22	
# H bonds	4		3	
# salt bridges	0		0	
residues at interface				
		H bond/salt bridge[b]		H bond/salt bridge[b]
	**Pro45**		**Pro45**	
	**Ala46**		**Ala46**	
	**Gln48**		**Gln48**	
	**Ala70**		**Ala70**	
	**Asn71**		**Asn71**	
	**Lys119**		**Lys119**	
	**Leu170**		**Leu170**	
	**Cys171**		**Cys171**	
			**Glu239**	
	**Glu240**		**Glu240**	
	**Leu242**		**Leu242**	
	**Lys243**		**Lys243**	
	**Ser246**		**Ser246**	
	**Thr247**		**Thr247**	
	**Ser276**		**Ser276**	
	**Glu277**		**Glu277**	
			**Val278**	
			**Glu279**	
	**Asn280**	H	**Asn280**	H
	**Lys281**		**Lys281**	
	**Asn282**	H	**Asn282**	H
	**Val283**		**Val283**	
	**Pro284**		**Pro284**	
	Glu287		Glu287	
	Pro289		Pro289	
	Glu290		Glu290	
	His291		His291	
	Gln294		Gln294	
	Glu295		Glu295	
	Glu296		Glu296	
	Lys299		Lys299	
	Ile319		Ile319	

	Pro320		Pro320	
	Gln324		Gln324	
	Tyr325		Tyr325	
	Arg460		Arg460	
	Asp462		Asp462	
	Leu463		Leu463	
	Met466		Met466	
	Asp469		Asp469	
	Lys470	H	Lys470	H
	Arg472	H	Arg472	H
	Glu474	H	Glu474	
	Asn475		Asn475	H
			Glu507	
		**interface 5**[a,b]		
	wild type		E111F mutant	
surface area (Å^2)	513/568		535/604	
number residues (primary/other molecule)	21/16		24/17	
# H bonds	2		3	
# salt bridges	0		0	
		residues at interface		
		H bond/salt bridge[b]		H bond/salt bridge[b]
	Met42		Met42	H
	Asn43		Asn43	
	Asn44	H	Asn44	H
	Pro45		Pro45	
	Ala46		Ala46	
	Ile50		Ile50	
	Asp52		Asp52	
	His53		His53	
	Arg65		Arg65	
	Ser263		Ser263	
	Asp265		Asp265	
			Asp266	
			Thr268	
	Asn269		Asn269	

	Val272		Val272	
	Lys273			
	Ser276	H	Ser276	H
	Glu277		Glu277	
	**Leu301**		**Leu301**	
	**Lys303**		**Lys303**	
			**Arg311**	
	**Glu381**		**Glu381**	
	**Glu382**		**Glu382**	
	**Leu384**		**Leu384**	
	**Leu385**		**Leu385**	
	**His386**		**His386**	
	**Val387**		**Val387**	
	**Glu388**		**Glu388**	H
	**Ile391**		**Ile391**	
			**Val478**	
	**Ile480**		**Ile480**	
	**Lys483**		**Lys483**	
	**Ser484**	H	**Ser484**	H
	**Phe485**		**Phe485**	
	**Glu486**		**Glu486**	
	**Lys488**	H	**Lys488**	H
	**Thr489**		**Thr489**	
	**Lys501**		**Lys501**	
	**Glu503**		**Glu503**	
			**Ile505**	
	**Val509**		**Val509**	
	**Trp513**		**Trp513**	
**interface 6**[a,b]				
	wild type		E111F mutant	
surface area (Å^2)	481/500		500/512	
number residues (primary/other molecule)	18/14		19/14	
# H bonds	5		5	
# salt bridges	1		1	
residues at interface				

		H bond/salt bridge[b]		H bond/salt bridge[b]
	Lys542		Lys542	
	Asp543		Asp543	HS
			**Ala544**	
			**Thr545**	
	**Pro546**		**Pro546**	
	**Tyr547**		**Tyr547**	
	Ile551		Ile551	
	Lys552		Lys552	
	Thr554		Thr554	
			**Asp565**	
	Lys735	HS	Lys735	H
	Gln736	H	Gln736	H
	Lys735	HS	Lys735	H
	Gln736	H	Gln736	H
	Leu739		Leu739	
	Gly740		Gly740	
	Gln743		Gln743	
	Met744		Met744	
	Asp747		Asp747	
	Glu751		Glu751	
	His752		His752	
	**Ser801**	H	**Ser801**	H
	**Thr802**		**Thr802**	
	**Ser803**	H	**Ser803**	H
	**Phe807**			
	**Asp919**		**Asp919**	
	**Asn922**		**Asn922**	
	**Ile923**		**Ile923**	
	**Ala926**		**Ala926**	
	**Tyr927**		**Tyr927**	H
	**Lys929**		**Lys929**	
	**Thr930**	H	**Thr930**	
	**Leu931**		**Leu931**	
	**Asp935**	HS	**Asp935**	
	**Lys938**		**Lys938**	HS
	**Phe939**			
	**Glu942**		**Glu942**	
interface 7[a,b]				
	wild type		E111F mutant	
surface area	289/279		422/424	

(Å^2)				
number residues (primary/other molecule)	12/12		15/16	
# H bonds	1		4	
# salt bridges	0		3	
residues at interface				
		H bond/salt bridge[b]		H bond/salt bridge[b]
	His53		His53	S
			Lys56	
	Pro58		Pro58	
	Glu59			
	Glu408		Glu408	
	Gln412		Gln412	H
			Lys415	S
			Asp416	H
	Val420		Val420	
	Arg423	H	Arg423	H
	Phe424		Phe424	
			Glu457	
			Glu458	
			Phe459	
	Thr533		Thr533	
	Asn534		Asn534	
	Phe535		Phe535	
	Glu536			
			**Arg782**	
	**Glu962**		**Glu962**	
	**Met963**		**Met963**	
			**Asp964**	S
			**Ser965**	H
	**Leu980**		**Leu980**	
	**Glu982**		**Glu982**	S
	**Pro985**		**Pro985**	
	**Leu986**	H	**Leu986**	H
	**Pro987**		**Pro987**	
	**Gln988**		**Gln988**	
	**Pro989**		**Pro989**	
	**Glu990**		**Glu990**	
	**Val991**		**Val991**	

	**His993**		**His993**	

[a] Interfaces were characterized with the program PISA [Krissinel E, Henrick K (2007) Inference of macromolecular assemblies from crystalline state. J Mol Biol 372: 774-797].

[b] There are seven unique contacts in the IDE crystals. Interface 1 is the dimer interface, which has been characterized separately (Table S2). Interface 2 occurs at a crystallographic symmetry element, with both molecules therefore contributing the same residues. Interfaces 3-7 do not occur at symmetry element positions, and the interfaces differ for the two molecules involved. Surface area and number of residues are given first for the primary molecule and then the other molecule involved in the interface. Residues from the primary molecule are highlighted in bold. Residues from the other molecule involved in the interface are in plain text.

[c] Only the type, not the number, of interactions is indicated.
